# Oral and Maxillofacial Surgery: Current Updates and Perspectives

**DOI:** 10.3390/jcm15010356

**Published:** 2026-01-03

**Authors:** Alessandro Antonelli

**Affiliations:** School of Dentistry, Department of Health Sciences, Magna Graecia University of Catanzaro, 88100 Catanzaro, Italy; alessandro.antonelli@unicz.it

## 1. Introduction

In recent decades, oral and maxillofacial surgery (OMFS) has experienced a remarkable evolution, driven by the rapid development of new technologies, refined surgical techniques, and the integration of patient-specific clinical and radiological data. Advances in imaging, digital planning, biomaterials, and minimally invasive procedures have profoundly reshaped traditional surgical approaches, improving both precision and patient outcomes. The availability of three-dimensional imaging, virtual surgical planning, and patient-specific prosthetic solutions has enabled more personalized treatments, reshaping both routine clinical care and complex reconstructive procedures [1,2,3].

## 2. Discussion

One of the most evident drivers of change is the deep integration of advanced imaging and digital workflows into daily practice. Three-dimensional diagnostics and virtual surgical planning are no longer confined to highly specialized centers but have become essential tools across orthodontics, orthognathic surgery, implantology, and reconstruction. Digital outcome assessment in dentofacial deformities exemplifies how digital planning directly enhances precision and predictability [4]. The connection between clinical scenarios and imaging investigations strengthens this paradigm, including the analysis of osseous landmarks relevant to inferior alveolar nerve block procedures, which highlights critical anatomical variability with immediate implications for anesthetic safety [5]. This concept is further explained in a CBCT–histopathologic correlation study that shows a rare large-scale epidemiological snapshot of oral and maxillofacial lesions, reinforcing the diagnostic value of three-dimensional imaging in real-world clinical settings [6].

Beyond conventional imaging, innovative approaches based on artificial intelligence (AI) are rapidly gaining traction in oral and maxillofacial imaging. A systematic review of AI-driven tooth segmentation in CBCT imaging emphasizes a crucial synthesis of algorithms, performance measures, and clinical applicability, demonstrating the rapid integration of automated diagnosis into everyday practice [7]. Together, these studies show how diagnostic practice is gradually moving from manual interpretation toward data-driven and partially automated decision-making.

Parallel to digital innovation, regenerative medicine and biologically oriented therapies continue to consolidate their role in OMFS. The growing clinical application of autologous platelet concentrates and bioactive biomaterials reflects a broader transition toward biologically assisted healing. This concept is supported by strong evidence on the regenerative potential of platelet-rich fibrin and related derivatives [8]. At the same time, the continuous refinement of dental implant surface and biomechanical optimization—as demonstrated by recent in vitro and clinical studies on implant stability—confirms how dental materials and biology are linked to gain more predictable results in implant rehabilitation [9,10,11].

Furthermore, innovation is not limited to digital technology and biomaterials, but also involves the development of new clinical protocols to manage the most complex conditions in OMFS. Of particular relevance is the report on the surgical management of oro-nasal communication in cocaine-induced midfacial destruction, treated through a temporalis muscle flap combined with Le Fort I osteotomy [12]. Beyond its technical value, this contribution addresses a growing and socially relevant clinical problem, where substance abuse intersects with severe patient morbidity. It also exemplifies how classical reconstructive principles remain indispensable when applied within contemporary multidisciplinary care pathways.

Moreover, OMFS oncology continues to represent one of the core domains of the discipline. In an umbrella review comparing conservative and radical approaches in the treatment of ameloblastoma [13], the authors showed a high-level evidence synthesis addressing one of the most debated dilemmas in the management of this tumor: the balance between local disease control and preservation of function and quality of life. Such contributions emphasize how decision-making in OMFS increasingly relies on outcome-weighted, patient-centered paradigms rather than purely ablative logic.

Trauma surgery, historically central to OMFS, is revisited through a population-wide perspective. The nationwide analysis of maxillofacial trauma trends in Germany spanning 2005–2022 provides a rare long-term view of epidemiologic shifts, injury mechanisms, and healthcare system burden [14]. These data not only inform clinical organization and resource allocation but also reinforce the preventive role of OMFS within public health strategies.

Finally, in an era of rapid technological convergence, the future of oral and maxillofacial surgery will increasingly rely on the integration of biologically driven therapies, digital innovation, and personalized surgical strategies, ultimately redefining patient-centered care and expanding the therapeutic boundaries of the discipline [15,16,17,18,19,20].

## 3. Conclusions

What clearly emerges is that OMFS can no longer be viewed as a purely technical surgical discipline but now stands at the intersection of digital medicine, regenerative biology, oncology, biomechanics, artificial intelligence, and population health. As a result, the field is becoming increasingly precise, biologically oriented, and data-driven.

Overall, this integrated perspective offers a forward-looking view of contemporary oral and maxillofacial surgery. By combining digital innovation, biologically assisted therapies, oncologic evidence, reconstructive complexity, and trauma epidemiology, it provides a valuable reference framework for clinicians, researchers, and trainees.

## Abbreviations

The following abbreviations are used in this manuscript:

OMFS	Oral and Maxillofacial Surgery

## References

[1] Alkhayatt N.M., Alzahrani H.H., Ahmed S., Alotaibi B.M., Alsaggaf R.M., Alalmuaysh A.M., Alomair A.A. (2024). Computer-assisted navigation in oral and maxillofacial surgery: A systematic review. Saudi Dent. J..

[2] Yeo X.H., Uei L.J., Yi M., Kungsadalpipob K., Subbalehka K., Al-Nawas B., Mattheos N. (2025). Computer-Assisted Implant Surgery: Patients’ Experience and Perspectives. Clin. Exp. Dent. Res..

[3] Sivolella S., Brunello G., Panda S., Schiavon L., Khoury F., Del Fabbro M. (2022). The Bone Lid Technique in Oral and Maxillofacial Surgery: A Scoping Review. J. Clin. Med..

[4] Barone S., Cosentini G., Bennardo F., Antonelli A., Giudice A. (2022). Incidence and management of condylar resorption after orthognathic surgery: An overview. Korean J. Orthod..

[5] Urîtu A., Roi C., Roi A., Motofelea A.C., Badea I., Chioran D., Riviș M. (2025). Imaging Retrospective Study Regarding the Variability of the Osseous Landmarks for IAN Block. J. Clin. Med..

[6] Alzahrani S., Wazzan T., Almaghrabi A., Alkhudran A., Aljereb H., Elsayed S., Alolayan A.B. (2024). The Prevalence and Diagnostic Patterns of Oral and Maxillofacial Lesions: A Seven-Year, Retrospective, Single-Center Cone Beam Computed Tomography and Histopathology Study in Saudi Arabia. J. Clin. Med..

[7] Tarce M., Zhou Y., Antonelli A., Becker K. (2024). The Application of Artificial Intelligence for Tooth Segmentation in CBCT Images: A Systematic Review. Appl. Sci..

[8] Miron R.J., Estrin N.E., Ahmad P., Farshidfar N., Fujioka-Kobayashi M., Zhang Y., Romandini M., Gruber R. (2025). Thirty Years of Autologous Platelet Concentrates: From Platelet-Rich Plasma to Platelet-Rich Fibrin. J. Periodontal. Res..

[9] Ayub F.A., Sunarso S., Dewi R.S. (2025). The Influence of Implant Macro-geometry in Primary Stability in Low-Density Bone: An in vitro Study. J. Int. Soc. Prev. Community Dent..

[10] Antonelli A., Barone S., Attanasio F., Salviati M., Cerra M.G., Calabria E., Bennardo F., Giudice A. (2023). Effect of Implant Macro-Design and Magnetodynamic Surgical Preparation on Primary Implant Stability: An In Vitro Investigation. Dent. J..

[11] Rittipakorn P., Ouyyamwongs W., Boonpitak K. (2025). Innovation of clockwise osseodensification technique for primary stability in dental implant: A low-density bone cadaveric study. Front. Dent. Med..

[12] Lupi E., Ciccozzi A., Becelli R., Mannino M., Bernardi S., Giovannetti F. (2025). Surgical Management of Oro-Nasal Communication in Cocaine-Induced Lesions: Temporalis Muscle Flap with Le Fort I Osteotomy. J. Clin. Med..

[13] Gasparro R., Giordano F., Campana M.D., Aliberti A., Landolfo E., Dolce P., Sammartino G., di Lauro A.E. (2024). The Effect of Conservative vs. Radical Treatment of Ameloblastoma on Recurrence Rate and Quality of Life: An Umbrella Review. J. Clin. Med..

[14] Meisgeier A., Pienkohs S., Dürrschnabel F., Moosdorf L., Neff A. (2024). Epidemiologic Trends in Maxillofacial Trauma Surgery in Germany-Insights from the National DRG Database 2005–2022. J. Clin. Med..

[15] Neff A.M. (2025). Editorial: Updates and Challenges in Maxillofacial Surgery. J. Clin. Med..

[16] Miragall M.F., Knoedler S., Kauke-Navarro M., Saadoun R., Grabenhorst A., Grill F.D., Ritschl L.M., Fichter A.M., Safi A.-F., Knoedler L. (2023). Face the Future-Artificial Intelligence in Oral and Maxillofacial Surgery. J. Clin. Med..

[17] Sillmann Y.M., Monteiro J.L.G.C., Eber P., Baggio A.M.P., Peacock Z.S., Guastaldi F.P.S. (2025). Empowering surgeons: Will artificial intelligence change oral and maxillofacial surgery?. Int. J. Oral Maxillofac. Surg..

[18] Mohaideen K., Negi A., Verma D.K., Kumar N., Sennimalai K., Negi A. (2022). Applications of artificial intelligence and machine learning in orthognathic surgery: A scoping review. J. Stomatol. Oral Maxillofac. Surg..

[19] Nicolae C.-L., Pîrvulescu D.-C., Niculescu A.-G., Epistatu D., Mihaiescu D.E., Antohi A.M., Grumezescu A.M., Croitoru G.-A. (2024). An Up-to-Date Review of Materials Science Advances in Bone Grafting for Oral and Maxillofacial Pathology. Materials.

[20] Zoabi A., Redenski I., Oren D., Kasem A., Zigron A., Daoud S., Moskovich L., Kablan F., Srouji S. (2022). 3D Printing and Virtual Surgical Planning in Oral and Maxillofacial Surgery. J. Clin. Med..

